# Surfactant-free synthesis of octahedral ZnO/ZnFe_2_O_4_ heterostructure with ultrahigh and selective adsorption capacity of malachite green

**DOI:** 10.1038/srep25074

**Published:** 2016-05-04

**Authors:** Jue Liu, Min Zeng, Ronghai Yu

**Affiliations:** 1 School of Materials Science and Engineering, Beihang University, Beijing 100191, China

## Abstract

A new octahedral ZnO/ZnFe_2_O_4_ heterostructure has been fabricated through a facile surfactant-free solvothermal method followed by thermal treatment. It exhibits a record-high adsorption capacity (up to 4983.0 mg·g^−1^) of malachite green (MG), which is a potentially harmful dye in prevalence and should be removed from wastewater and other aqueous solutions before discharging into the environment. The octahedral ZnO/ZnFe_2_O_4_ heterostructure also demonstrates strong selective adsorption towards MG from two kinds of mixed solutions: MG/methyl orange (MO) and MG/rhodamine B (RhB) mixtures, indicating its promise in water treatment.

Nowadays, it is imperative to avoid water contamination due to the residual organic dyes in industrial and agricultural effluents that pose potential threat to environment or human health for their wide application^1^,^2^,^3^,^4^,^5^,^6^. As is known, decomposition of the organic dyes is of exceptional difficulty under natural condition owing to its complex and steady chemical structure. Methods have been reported for effective elimination of the recalcitrant dyestuff from aqueous solutions, including physical, chemical, and biological approaches such as volatilization, electrochemical treatment, hydrolysis, photolysis, oxidation, biodegradation, adsorption and so on, among which the adsorption process is prevailing due to its simplicity and high efficiency^1^,^2^,^3^,^4^,^7^,^8^,^9^,^10^,^11^,^12^,^13^,^14^,^15^,^16^,^17^,^18^,^19^,^20^. Porous and nanostructured materials have attracted considerable attention as promising adsorption candidates, including activated carbon^21^, zeolite^22^, clay^23^, modification diatomite^24^, boron nitride^17^,^25^ and graphene^26^,^27^ *et al.* Materials possessing high porosity, specific surface area and permeability can combine with contaminants and offer the possibility of being enriched with the target pollutants^28^,^29^,^30^,^31^,^32^. Additionally, some polymers can be represented as an interesting alternative^11^,^15^, for their effective removal of toxic dyes from aqueous solution.

Malachite green (MG), a cationic triphenylmethane dye in high prevalence as a biological stain or therapeutic agent, is also involved in severe controversy for the risks of its tumorigenicity, carcinogenicity, mutagenicity and teratogenecity^33^,^34^,^35^. Nevertheless, it can hardly be banned completely considering its cost effectiveness, commercial availability and high efficiency. Meanwhile, the presenting adsorbents towards MG still suffer from limited removal capacity. In addition, in order to meet the growing demand of complex dye-containing wastewater treatment, sometimes removal of all the dyes is not always necessary since some valuable chemicals in the discharge streams need to be recycled^11^,^12^,^13^,^14^,^15^,^36^,^37^,^38^,^39^,^40^,^41^.

Recently, supramolecular hydrogels^41^, magnetic nanoparticles^19^,^36^ and metal-organic frameworks (MOFs)^8^,^14^,^37^,^38^,^39^,^40^,^42^ have generated increasing interest as dye adsorbents because of their high removal capacity, selective adsorption and separation. The selectivity is usually realized by non-covalent host–guest interactions such as π–π interaction, electrostatic interaction, hydrogen bonding, H-bonding, p–p stacking, hydrophobic interaction, and acid-base interaction between the adsorbent and dye molecules.

Several ZnO/ZnFe_2_O_4_ structures have been explored as novel functional materials^43^,^44^, and even with promising application in water treatment^45^. Nevertheless, construction of efficient adsorbent materials offering the possibility of fast and high selective adsorption towards MG has not been fully developed so far. With this consciousness to realize such an advanced adsorbent, a new ZnO/ZnFe_2_O_4_ heterostructure as the outcome via rational design and facile synthesis demonstrates a record-high uptake capacity, evidenced by many pioneering works (listed in Supplementary Table S1), and selective removal of MG from mixed solutions.

## Results

### Preparation of octahedral ZnO/ZnFe_2_O_4_ heterostructures

**Figure 1:**
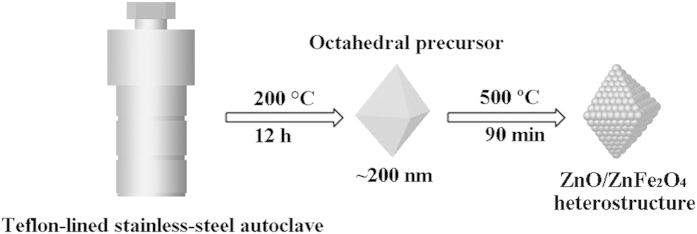
Schematic illustration for the preparation process of the Octahedral ZnO/ZnFe_2_O_4_ heterostructures.

The ZnO/ZnFe_2_O_4_ nanoparticles were synthesized in a two-step procedure (illustrated in Fig. 1). First, we put forward a simple solvothermal methodology for preparation of precursor without any surfactant added. The morphology of the as-obtained light grey product is studied by scanning electron microscopy (SEM). The SEM image consists of many octahedral architectures with an average size of about 200 nm (Supplementary Figure S1). A series of other measurements are also used to investigate the structure of the precursor. The X-ray diffraction (XRD) pattern (Supplementary Figure S2) shows the emergence of a strong peak located in the low-angle region (about ca. 10°), exhibiting a similarity to those of other metallic organic structures reported in the literature^43^,^44^, though the exact crystal structure is not yet determined. In the Fourier transform IR (FTIR) spectra (Supplementary Figure S3), the adsorption peaks at the wavenumbers 2884 cm^−1^ and 2844 cm^−1^ can be attributed to the stretching vibration of –CH– groups. At the wavenumber 1120 cm^−1^ and 1079 cm^−1^ are the stretching vibration peaks of –C–O– groups. Thus, we concluded that the as-synthesized precursor may be a kind of metallic organic structures.

**Figure 2:**
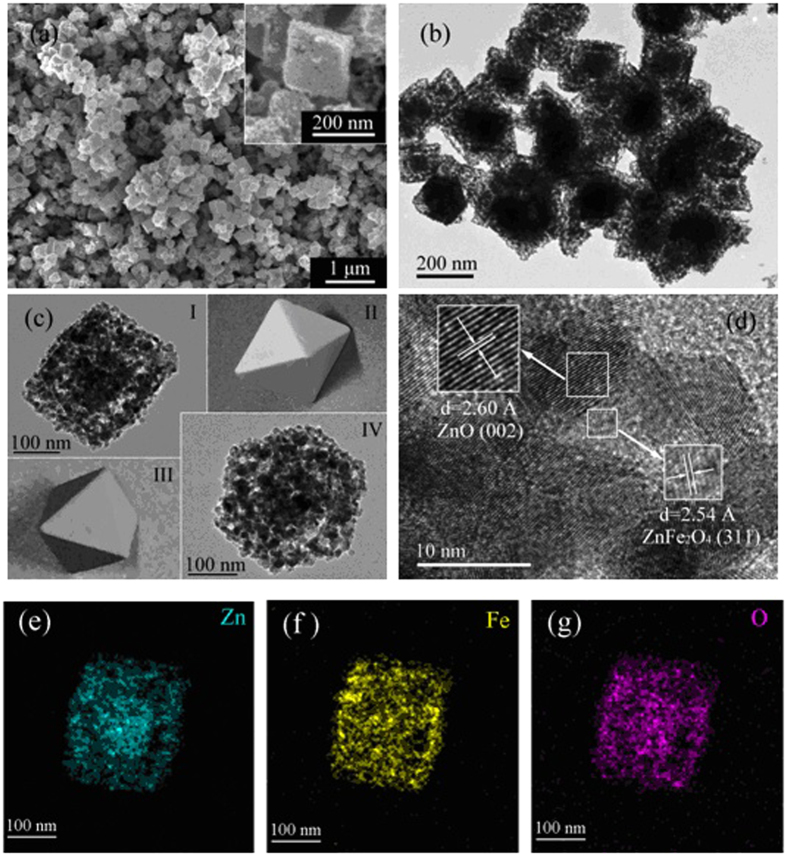
(**a**) FESEM, inset is a high solution image of a particle. (**b**) FETEM image and (**c**) FETEM images of a particle from different perspectives. (**d**) HRTEM image of the ZnO/ZnFe_2_O_4_ nanoparticles. EDS elemental mapping images to the corresponding area: (**e**) Zn, (**f**) Fe and (**g**) O.

Thermogravimetric (TG) analysis confirms that the organic molecules are escaped near 350 °C in air (Supplementary Figure S4). It shows a significant weight loss of 34% at 500 °C. The conversion from the precursor to the resulting octahedral ZnO/ZnFe_2_O_4_ heterostructures is realized via a moderate annealing treatment at 500 °C for 90 min in air with a ramping rate of 1 °C·min^−1^ from room temperature. The morphology of the octahedral structure is maintained after the thermal treatment (Fig. 2a). The obtained sample is found to own good uniformity with an average size of approximately 200 nm, in accord with the precursor (Supplementary Figure S1). The inset in Fig. 2a displays a higher magnification SEM image with a rough surface, resulting in high porosity that contributes to an enhanced adsorption capacity. TEM observations show a more porous structure, which might be generated by gas products escaping from the precursor during thermal decomposition process (Fig. 2b). The closer inspection provides further insight into the structural information of the porous octahedra. In Fig. 2c, Image I, Image IV are the TEM scannograms from different perspectives of a same octahedral particle, photo-illustrated as Image II and Image III, respectively. Also, it can be clearly seen that the octahedra are composed of many ultrafine nanoparticles with an average diameter of around 10 nm.

As observed in the HRTEM image (Fig. 2d), clear lattice fringes can be seen obviously, in which the 2.60 Å can be assigned to the (002) interplane spacing of ZnO, and the 2.54 Å belongs to the (311) interplane spacing of ZnFe_2_O_4_. This observation strongly suggests that ZnO and ZnFe_2_O_4_ nanoparticles are attached with each other and well dispersed in various orientations. The elemental mapping by energy dispersive spectroscopy indicates the uniform distribution of Zn, Fe and O species in the sample. (Fig. 2e,f).

**Figure 3:**
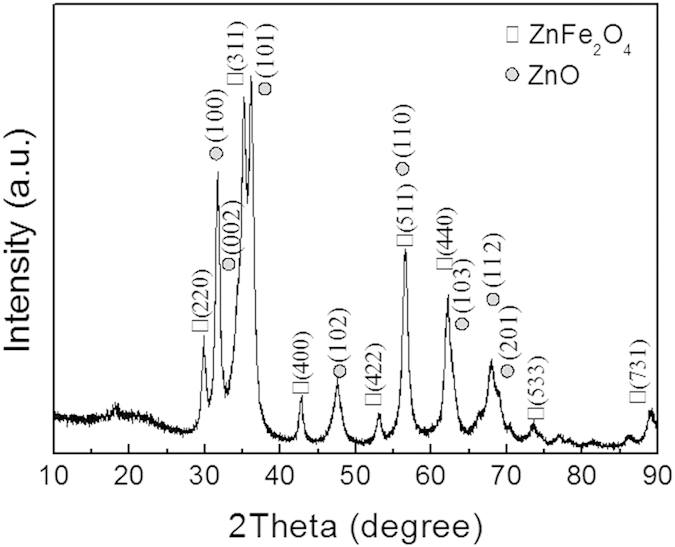
XRD pattern of the ZnO/ZnFe_2_O_4_ nanoparticles.

Figure 3 reveals the X-ray diffraction pattern of the as-prepared ZnO/ZnFe_2_O_4_ octahedra. It can be seen that the sample is of great crystallinity and composed of hexagonal ZnO (PDF#89-0510, P63mc(186)) and cubic ZnFe_2_O_4_ (PDF#74-2397, Fd-3m(227)).

**Figure 4:**
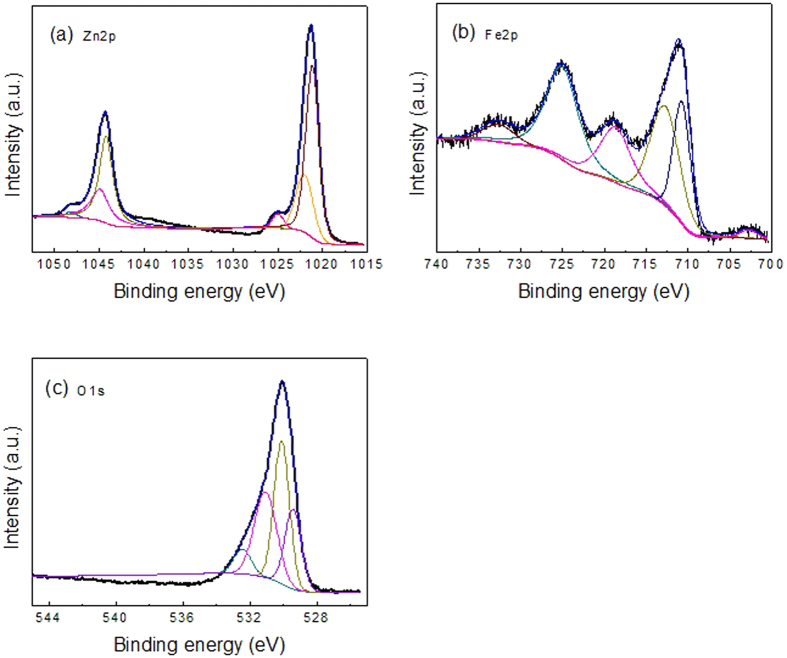
XPS analysis for the ZnO/ZnFe_2_O_4_ nanoparticles: (**a**) Zn 2p, (**b**) Fe 2p and (**c**) O 1s.

In order to examine the existence state of the elements in the as-synthesized ZnO/ZnFe_2_O_4_ octahedra, X-ray photoelectron spectra (XPS) measurements are carried out and the corresponding fitted data on the resultant product in the Zn, Fe and O region are demonstrated in Fig. 4a–c, respectively. In Fig. 4a for Zn 2p spectrum, the fitting peaks at 1044.98 and 1022.05 eV are related to Zn 2p^1/2^ and Zn 2p^3/2^, respectively, indicating the existence state of Zn element in ZnFe_2_O_4_ is Zn^2+^. The peaks at 1044.23 and 1021.16 eV are attributed to divalent Zn belonging to ZnO. The fitting peaks of 712.87 and 710.77 eV for Fe 2p^3/2^ spectrum in Fig. 4b are ascribed to tetrahedral and octahedral sites of ZnFe_2_O_4_ respectively. The peaks at 724.97 and 718.72 eV are of Fe 2p^1/2^ spectrum, indicating the state of Fe in the sample is Fe^3+^. For O 1s spectra in Fig. 4c, the peak at 529.47 eV is assigned to typical lattice oxygen in the Zn–O or Fe–O structure. The peak at 530.14 eV indicates the O element from other components absorbed on the surface of the sample. In addition, the peak at 532.48 eV is originated in the defect of low oxygen co-ordination in the nanostructure^43^.

**Figure 5:**
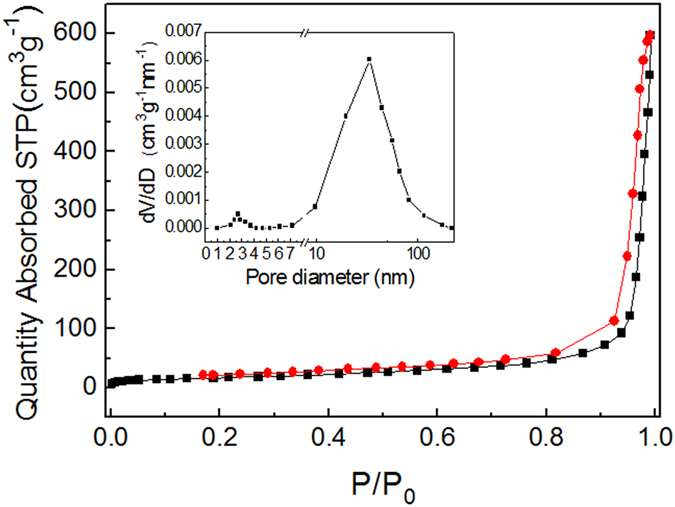
N_2_ adsorption-desorption isotherm for the ZnO/ZnFe_2_O_4_ nanoparticles (inset is the pore size distribution).

To further investigate the specific surface area and the porous nature of the product, Brunauer-Emmett-Teller (BET) gas-sorption measurements were performed at 77 K. Fig. 5 shows the N_2_ adsorption-desorption isotherms at 77 K and the corresponding Barrett-Joyner-Halenda (BJH) pore-size distribution. The as-synthesized ZnO/ZnFe_2_O_4_ product possesses a BET specific surface area of 60.45 m^2^·g^−1^ and a total pore volume of 0.94 cm^3^·g^−1^ with an average pore size of about 39.9 nm, calculated from the desorption branch. In the thermal treatment process, elimination of organic groups which converse the precursor into co-existing ZnO and ZnFe_2_O_4_ nanostructures leaves pores with a relatively broad distribution behind, which may play vital roles in the selective adsorption of different-sized dye molecules.

### MG adsorption

**Figure 6:**
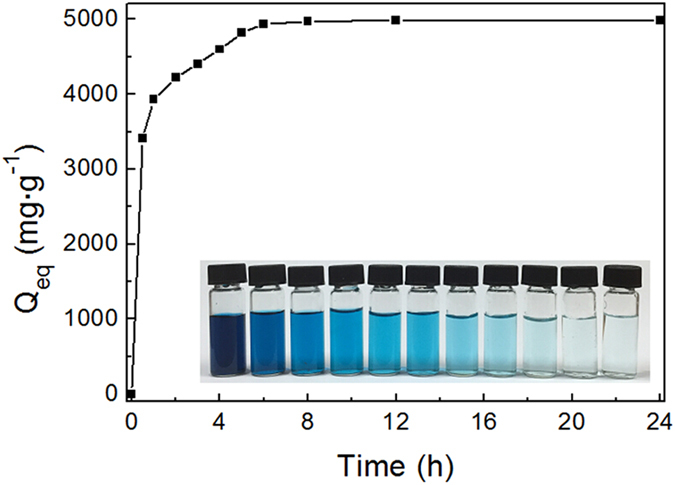
Adsorption capacity of the ZnO/ZnFe_2_O_4_ nanoparticles towards MG at various contact time. Inset: the color change of the MG solution at given intervals.

To evaluate the maximum adsorption behavior of ZnO/ZnFe_2_O_4_ nanostructures, 5 mg samples are exposed to 50 mL MG aqueous solution (0.5 g·L^−1^). To ensure that the adsorption equilibrium is reached, the dye concentrations are recorded for 24 h. The digital images show that the solutions become colorless gradually (inset in Fig. 6). However, with a larger amount of MG aqueous solution, the final solution becomes less blue but not colorless.

The dye concentrations in these solutions are detected by a UV-visible spectroscopy illustrated in Supplementary Figure S5. The amount of adsorbed dye is calculated using Equation (1):


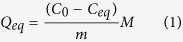


where *Q*_*eq*_ is the amount of dye adsorbed by adsorbent (mg·g^−1^), *C*_0_ is the initial concentration of dye (mmol·L^−1^), *C*_*eq*_ (mmol·L^−1^) is the concentration of dye at equilibrium, *V* (L) is the volume of solution, *M* (g·mol^−1^) is the molecular weight of the dye, *m* (g) is the mass of adsorbent that we soak.

Figure 6 shows the adsorption capacities for MG with different contact times. At the beginning, the adsorption capacity Q_eq_ increases significantly with the increasing time, but it gradually reaches an adsorption equilibrium. By calculations, the as-synthesized ZnO/ZnFe_2_O_4_ nanostructures have an adsorption capacity of 4983.0 mg·g^−1^ for MG, a record-high value that is much higher than that of the previously reported metal oxides, carbon, MOFs and other adsorbents as we have known (listed in Supplementary Table S1), with a removal percentage exceeded 99.5%. Therefore, the ZnO/ZnFe_2_O_4_ nanostructure with high uptake capacity for dye pollutant and facile and low-cost preparation process is favorable in wastewater treatment.

### Influence of the pore size on the adsorption ability

The pore size is an important factor for the adsorption ability. For comparison, we also explored the adsorption properties of ZnO/ZnFe_2_O_4_ nanostructures obtained by thermal annealing at 500 °C with a ramping rate of 5 °C·min^−1^ to trigger a different pore-size distribution. In Figure S7, the product (denoted as Sample 1) is as same as the sample (denoted as Sample 2) represented in Fig. 3, both with the existence of crystalline ZnO and ZnFe_2_O_4_. However, the pore-size distribution of Sample 1 shown in Figure S8 is different from that of Sample 2, leading the adsorption performance of Sample 1 worse than that of the well-defined ZnO/ZnFe_2_O_4_ octahedra (Seen from Figure S9).

### Adsorption kinetics

**Figure 7:**
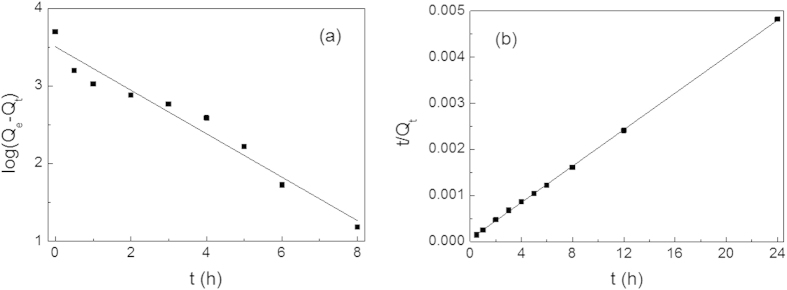
Adsorption kinetics based on (**a**) pseudo-first-order model and (**b**) pseudo-second-order model.

The transint rate-controlling steps involved in the adsorption process can be analyzed using the pseudo-first-order or pseudo-second-order models. The linearized pseudo- first-order kinetic model is given as the following equation^46^:


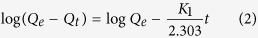


where *Q*_*e*_ (mg·g^−1^) is the amount of dye adsorbed at an equilibrium and *Q*_*t*_ (mg·g^−1^) is the amount of dye adsorbed at a certain time *t* (h), *k*_1_ (h^−1^) is the adsorption rate constant. As for pseudo-second-order kinetic model, it can be expressed as follows^46^:


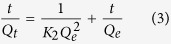


where *k*_2_ (g·mg^−1^·h^−1^) is the adsorption rate constant. The linear plots of log(Q_e_−Q_t_) vs. *t* for the pseudo-first-order and (*t*/*Q*_*t*_) vs. *t* for pseudo-second-order models are plotted in Fig. 7. The rate constants *k*_1_ and *k*_2_ are deduced by fitting the experimental data. The correlation coefficient (*R*^2^) is 0.9546 for pseudo-first-order model and 0.9997 for pseudo-second-order model, indicating the adsorption behavior follows the pseudo-second-order kinetic model rather than the other. Therefore, the adsorption rate depends on the amount of solute at equilibrium.

**Figure 8:**
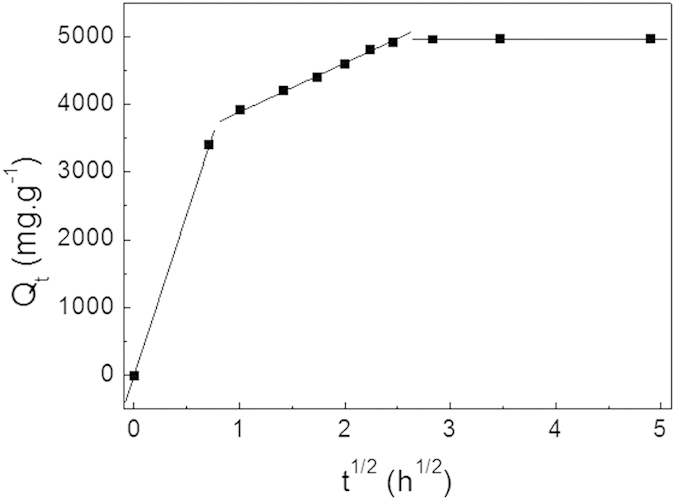
Intraparticle diffusion kinetic plot for the adsorption of MG into ZnO/ZnFe_2_O_4_ nanostructures.

Moreover, the pore-diffusion (intraparticle diffusion) model is presented to describe the adsorption process for a porous adsorbent. The uptake of the adsorbate is related to the square root of time^47^:


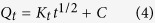


where *K*_*t*_ (mg·g^−1^·h^1/2^) is the intraparticle diffusion rate and *C* (mg·g^−1^) is a constant. In Fig. 8, the plots of *Q*_*t*_ against *t*^1/2^ show at least three linear stages in correspondence with different stages in adsorption. The line in the first stage nearly passes through the origin, indicating that the uptake is dominated by the intraparticle diffusion process. The second stage indicates a steady step, corresponding to diffusion of MG molecules to the pores. In the third phase, diffusion remains fairly constant when the pore volume is exhausted. Thus, the diffusion of MG into pores of ZnO/ZnFe_2_O_4_ nanostructures, as well as adsorption on the available surface is probably responsible for the adsorption.

### Adsorption isotherm

**Figure 9:**
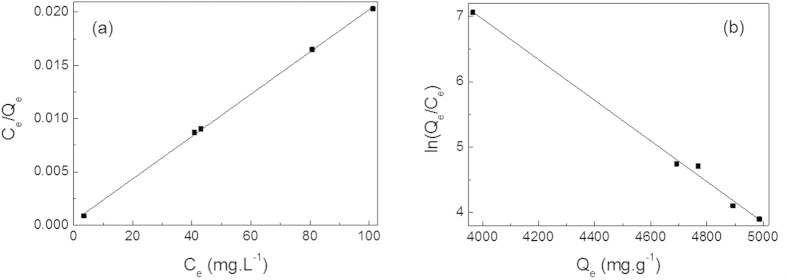
Fitting plots for the equilibrium adsorption based on (**a**) Langmuir isotherm model and (**b**) Freundlich isotherm model.

The equilibrium adsorption information can be interpreted using Langmuir^48^ or Freundlich models^49^:


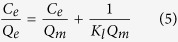



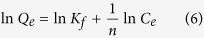


where *C*_e_ (mg·L^−1^) is the concentration of dye and *Q*e (mg·g^−1^) is the adsorption capacity at equilibrium, *K*_*l*_ (L·mg^−1^) is a affinity constant and *Q*_m_ (mg·g^−1^) is the maximum adsorption capacity. *K*_*f*_ ((mg·g^−1^)·(L·mg^−1^) ^−1^) is the Freundlich factor, and *n* is a constant correlated to maximum sorption capacity. The Langmuir isotherm model (Equation 5) assumes the adsorption process is a monolayer adsorption on a homogeneous adsorbent surface that the adsorption takes place at specific homogeneous sites within the adsorbent, while the Freundlich isotherm model (Equation 6) not restricted to the formation of a monolayer describes the adsorption on a heterogeneous surface with interaction between the adsorbed molecules. The fitting linear plots of *C*_e_/*Q*_e_ *vs. C*_e_ for Langmuir model and ln(*Q*_e_/*C*_e_) vs. *Q*_e_ for Freundlich model of the equilibrium adsorption at room temperature are shown as Fig. 9.

The *R*^*2*^ values of the Langmuir and Freundlich isotherm models are 0.9992 and 0.9373, respectively. The adsorption of MG molecules into the ZnO/ZnFe_2_O_4_ nanostructures follows the Langmuir isotherm rather than the later. The fitting results give high linearity of the correlation coefficient with Langmuir model, suggesting the dye molecular monolayer coverage of on the surface of ZnO/ZnFe_2_O_4_ nanostructures.

### Selective adsorption of MG from mixed-dye solutions

With an attempt to explore the selective adsorption potentiality for MG, a competitive experiment was performed. Since we know the fact that the ZnO/ZnFe_2_O_4_ nanostructures can adsorb MG effectively, two other organic dyes with different charges, structures and sizes, namely methyl orange (MO) and rhodamine B (RhB) are chosen to mix with MG, respectively. The temporal evolution of UV-vis spectra for the MO and RhB solutions are shown in Supplementary Figures S6 and S7, respectively, revealing rare MO and RhB are adsorbed. The chemical details of these organic dyes are presented in Supplementary Table S2. Both cationic MO and anionic RhB molecules are not adsorbed by the adsorbent, while anodic MG presents quite a different adherence. This evidence suggests that charge selective adsorption might not be the main reason for the adsorption.

**Figure 10:**
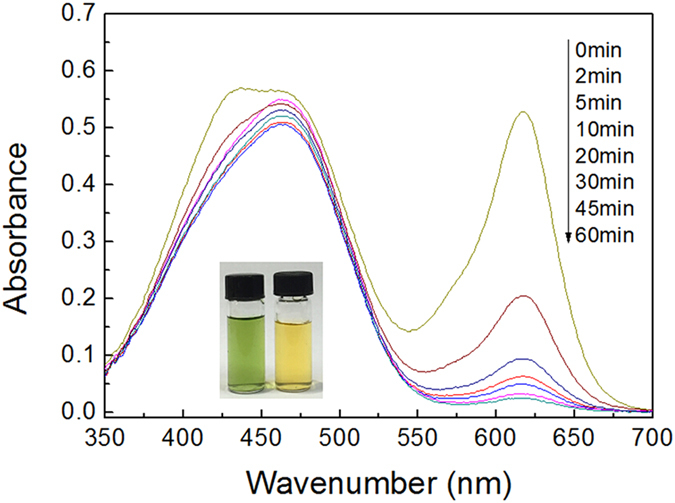
The selective adsorption of ZnO/ZnFe_2_O_4_ nanoparticles towards the mixed MG/MO solution. Inset: the color change of the mixed dyes solution before and after adsorption process.

The selective adsorption experiment is usually carried out using those dyes with different structural properties or charges. Here, two kinds of the mixtures are prepared. One consists of 25 mL MO (0.05 mmol·L^−1^) aqueous solution and 25 mL MG (0.05 mmol·L^−1^) aqueous solution, resulting in a green solution. Then, 5 mg of the adsorbent are added into the mixed solution to start the adsorption process. The dye solutions with different time intervals are detected by the UV-vis spectroscopy and shown in Fig. 10. At the beginning, the UV-vis spectrum of the solution has two main peaks at 465 nm (belonging to MO) and 665 nm (belonging to MG), respectively. Gradually, the peak of MG declines, while the peak of MO remains almost constant. In addition, the whole adsorption process happens within 60 min. The corresponding digital images before and after the adsorption process are showed in the inset of Fig. 10. Before adsorption, the solution has a green color, reflecting the co-existence of MO and MG. 60 min later, yellow solution, the original color of the MO solution, is observed.

**Figure 11:**
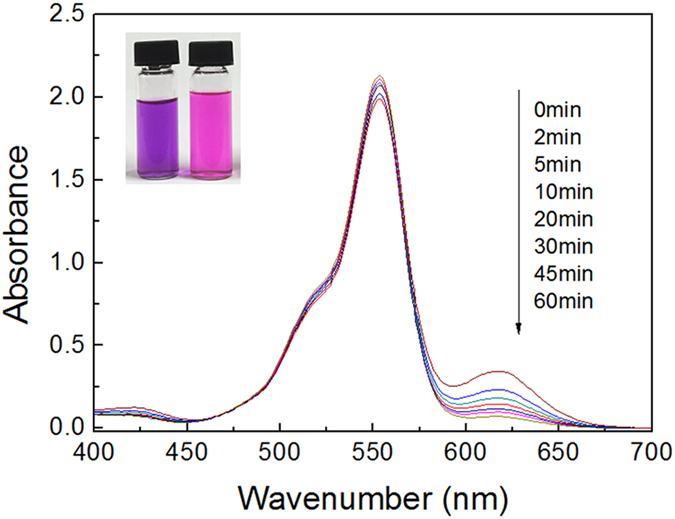
The selective adsorption of ZnO/ZnFe_2_O_4_ nanoparticles towards the mixed MG/RhB solution. Inset: the color change of the mixed dyes solution before and after adsorption process.

Subsequently, a similar experiment is carried out with a solution of mixed RhB and MG. The UV-vis spectra reveal the disappearance of MG signal, while the RhB absorbance peak at 553 nm only shows little decline (Fig. 11). Meanwhile, the color of MG fades, leaving the color of RhB remains unchanged (see from the inset in Fig. 11). The results indicate that the adsorbent can rapidly and selectively adsorb MG molecules rather than MO and RhB molecules.

### Mechanism of the selective adsorption

It has been clearly demonstrated that the ZnO/ZnFe_2_O_4_ nanostructures exhibit superior adsorption properties of MG dye molecule compared with MO and RhB dye molecules. It is also of significance to understand the mechanism of the selective adsorption behavior towards diverse organic dyes.

As mentioned above, the BET specific surface area of ZnO/ZnFe_2_O_4_ nanostructure is 60.45 m^2^·g^−1^, a normal value for which it is not possibly the main reason for the strong adsorption process towards MG. The ZnO/ZnFe_2_O_4_ nanostructures possess a negative Zeta potential of −6.4 mV. Besides, the negative charges on the surface of the adsorbent may cause them to adsorb cationic MG dye rather than anionic MO dye. However, this assumption cannot explain why it possesses negligible adsorption for cationic RhB dye. There should be another perspective needed to clarify the selective removal mechanism.

Then, a series of detailed studies have been performed to clarify the reason for the adsorption. The upper clear solution after adsorption is analyzed by ICP spectrometry for Zn and Fe. For MG (10 mL, 0.05 mmol·L^−1^ with 5 mg adsorbent), the content of Zn in upper clear solution is 12.57 mg·L^−1^. Interestingly, no Fe is detected. While in a richer MG solution (50 mL, 0.5 g·L^−1^ with 5 mg adsorbent), Zn reaches 41.29 mg·L^−1^ with none of Fe. As a result, the content of Zn increases with an increased adsorption capacity, suggesting the ion-exchange is probably the key for the occurrence of adsorption towards MG, where the free cations of Zn^2+^ rather than Fe^3+^ can be exchanged during the adsorption process. At the same time, for RhB and MO (10 mL, 0.05 mmol·L^−1^ with 5 mg adsorbent) the contents of Zn are 5.560 mg·L^−1^ and 3.470 mg·L^−1^, respectively, with no trace of Fe. For comparison, Zn in pure water is still detected with a content of 3.102 mg·L^−1^, similar to that of the MO solution. The fact indicates poor ion-exchange happens between MO and the adsorbent, which can also explain the poor adsorption performance. However, Zn content is also hither in the RhB solution than in water solution, which might be ascribed to the relatively easy interaction between RhB and the negative-charged adsorbent. But the RhB molecules are still negligible adsorbed, illustrating the uptake of the dye may be also determined by the matching property between the pore structure of the adsorbent and the size, steric shape of the dye molecule besides the ion-exchange.

Therefore, only those with acceptable sizes, matched charges and less steric hindrance may be exchanged into the void space of the structures, triggering a strong adsorption. The open metal sites and the suitable pore space are strongly suggested to play vital roles in remarkable storage of MG molecule under ambient conditions besides the ion-exchange. Nevertheless, the thorough removal mechanism remains indistinguishable due to it is very difficult to *in situ* monitor the adsorption process for many diverse dyes.

#### Synthesis

##### Synthesis of precursor

The precursor was synthesized by a solvothermal method. ZnCl_2_ (4.5 mmol) and FeCl_3_·6H_2_O (1.5 mmol) were dissolved in 30 ml ethylene glycol (EG), respectively. Then CH_3_COONa (1.2 g) was added into the mixed solution. The obtained mixture was stirred for 30 min to ensure dissolution, then sealed into a tightly Teflon-lined stainless-steel autoclave and maintained at 200 °C for 12 h. The obtained sample had a grey-white color and was washed with ethanol.

Synthesis of ZnO/ZnFe_2_O_4_ octahedra: after the precursor annealed in air at 500 °C for 90 min with a heating rate of 1 °C·min^−1^, final product was obtained.

#### Characterization

The X-ray diffraction (XRD) patterns were obtained with Rigaku D/max2200PC using CuKa radiation. The morphology were characterized using scanning electron microscopy (SEM, JSM-7500F). Transmission electron microscopy (TEM) images were obtained on a JEM-2100F transmission electron microscope. X-ray photoelectron spectroscopy (XPS) analysis was performed with an AEM PHI 5300 electron spectrometer using AlKa radiation. Ion concentrations were determined by inductively coupled plasma (ICP) atomic emission spectroscopy (Optima 7000DV, PerkinElmer). Thermogravimetric analysis (TGA) measurements were carried out in a temperature range from room temperature to 800 °C with a heating rate of 8 °C·min^−1^ under air flow. The Brunauer–Emmett–Teller (BET) surface area was determined by nitrogen adsorption using a V-Sorb 2800P Surface Area and Pore Distribution Analyzer. All UV-vis spectra were measured on a UV-vis spectrophotometer (Shimadzu, UV-3600).

#### Dye adsorption

For adsorption experiments hydrophilic dyes were chosen (Supplementary Table S2). The dyes were used and dissolved at a known concentration in distilled water. Then, the adsorbent was soaked into the dyes aqueous solution with incontinuous shaking at room temperature. The adsorption behavior was monitored by UV-vis spectroscopy, and the dye concentration was calculated using the maximum peak.

## Additional Information

**How to cite this article**: Liu, J. *et al.* Surfactant-free synthesis of octahedral ZnO/ZnFe_2_O_4_ heterostructure with ultrahigh and selective adsorption capacity of malachite green. *Sci. Rep.* **6**, 25074; doi: 10.1038/srep25074 (2016).

## Supplementary Material

Supplementary Information
