# Supplementary material for: Surfactant-free synthesis of octahedral ZnO/ZnFe2O4 heterostructure with ultrahigh and selective adsorption capacity of malachite green
Source: Sci Rep. 2016 May 4;6:25074. doi: 10.1038/srep25074 (PMC4855229; doi:10.1038/srep25074)
Supplement: Supplementary Information [file srep25074-s1.pdf]

Supplementary Information

Surfactant-free synthesis of octahedral ZnO/$ZnFe_2O_4$ heterostructure with ultrahigh and selective adsorption capacity of malachite green

Jue Liu[1], Min Zeng*[1] & Ronghai Yu*[1]

[1]School of Materials Science and Engineering, Beihang University, Beijing 100191, China. Correspondence and requests for materials should be addressed to M. Z. (min_zeng@buaa.edu.cn) or to R. Y. (rhyu@buaa.edu.cn).

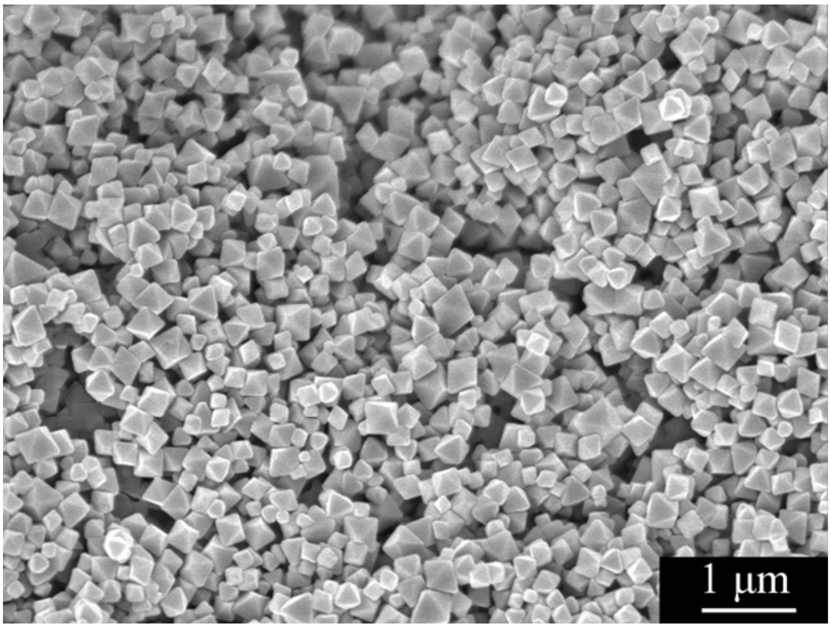

Figure S1 | SEM image of the octahedral precursor with an average size of about 200 nm.

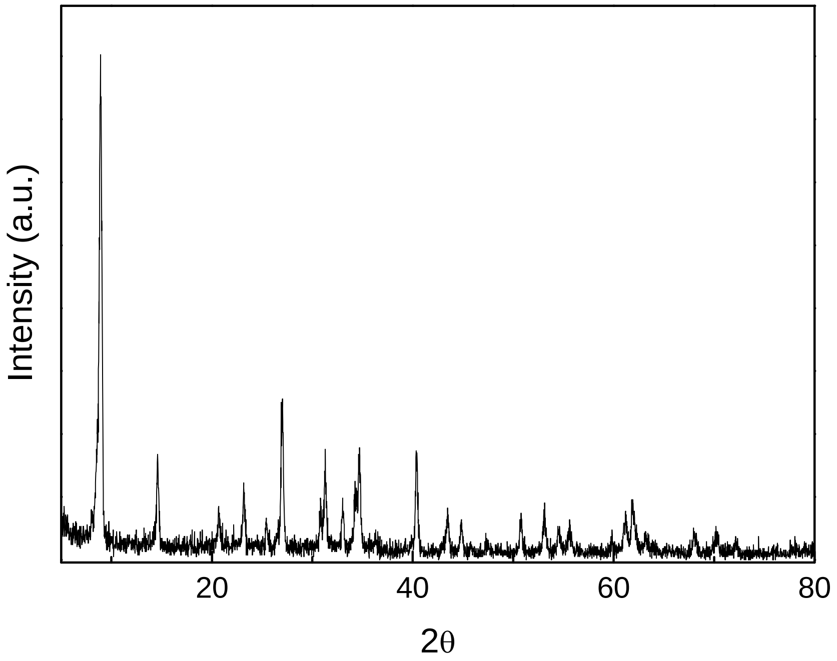

Figure S2 | XRD pattern of the octahedral precursor.

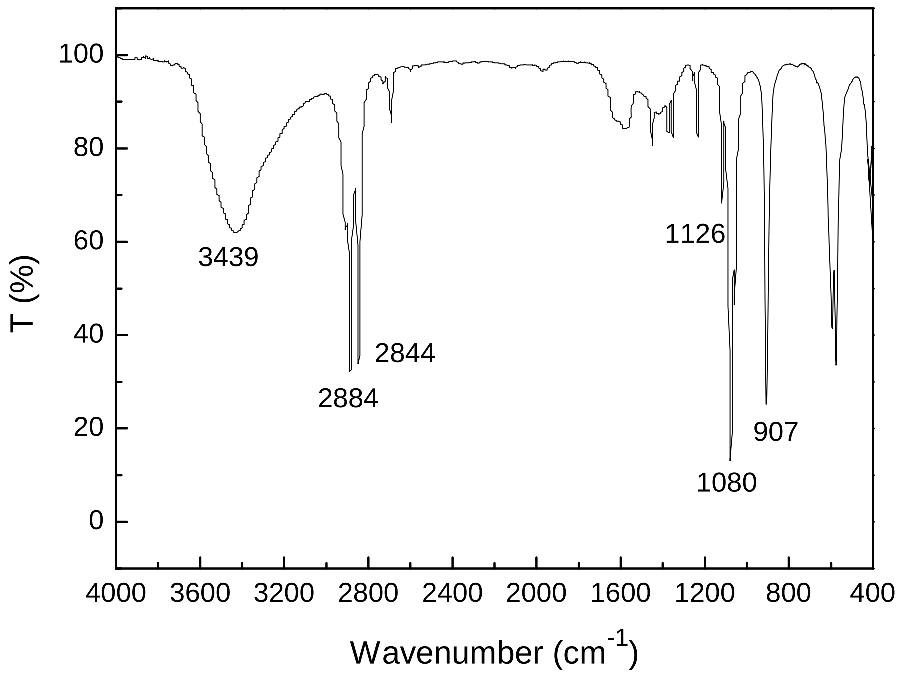

Figure S3 | Fourier transform IR (FTIR) spectra of the octahedral precursor.

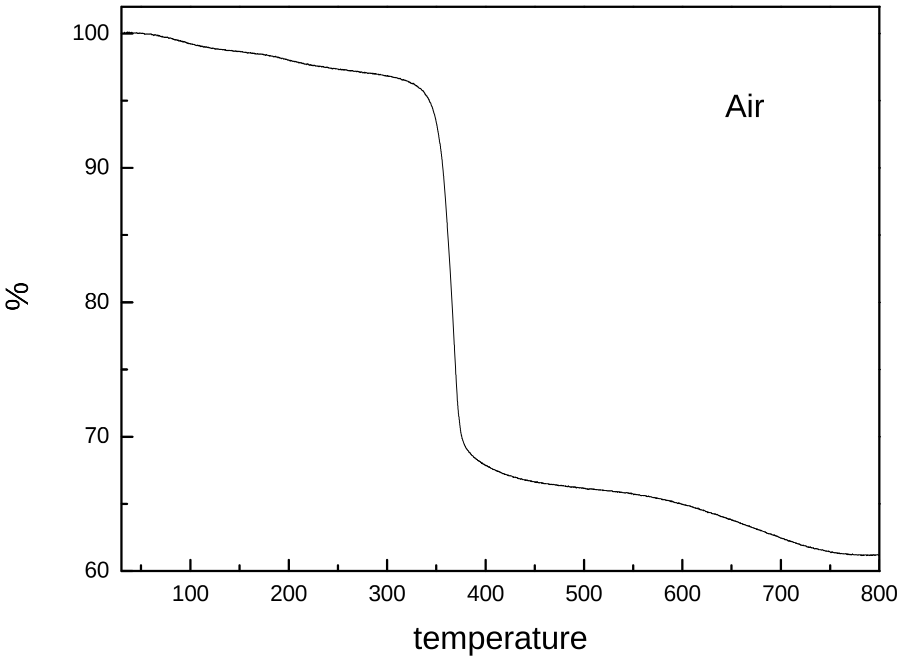

Figure S4 | TGA curve of the weight loss from room temperature to 800 °C.

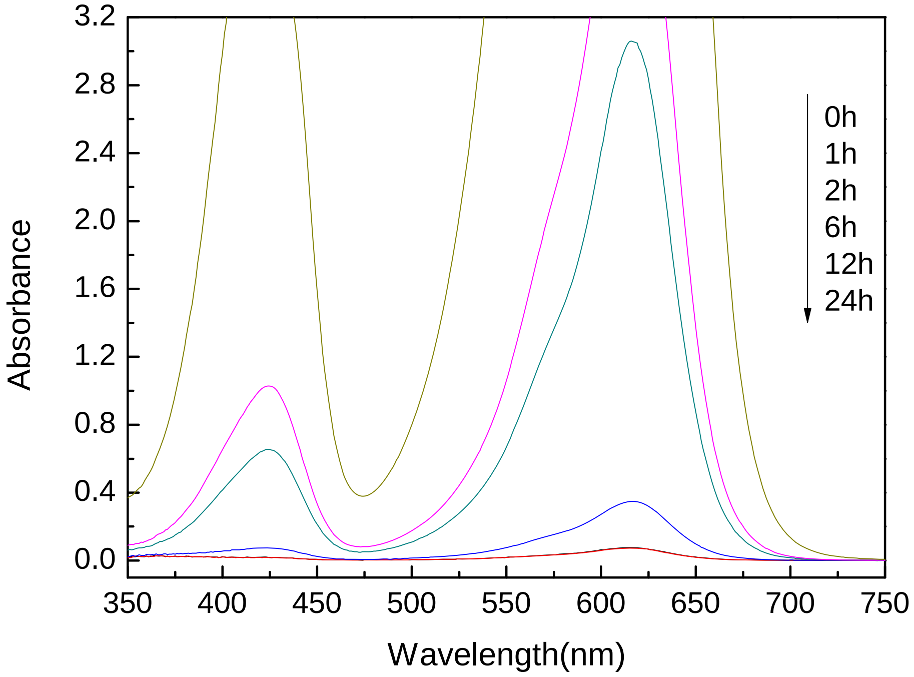

Figure S5 | The temporal evolution of UV spectra of MG solution (0.5 g·L^{-1})

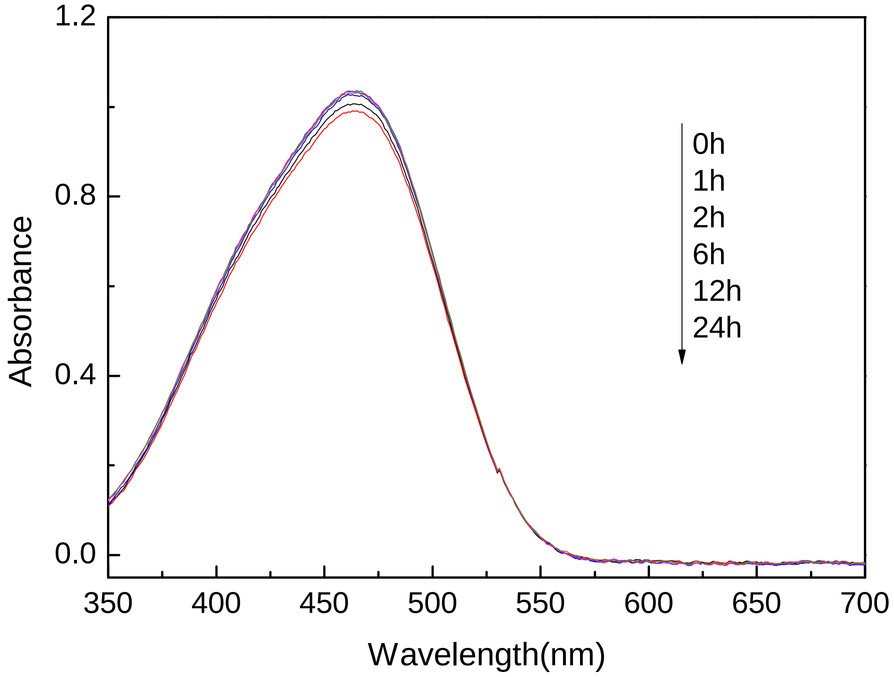

Figure S6 | The temporal evolution of UV spectra of MO solution (10 mg·L^{-1})

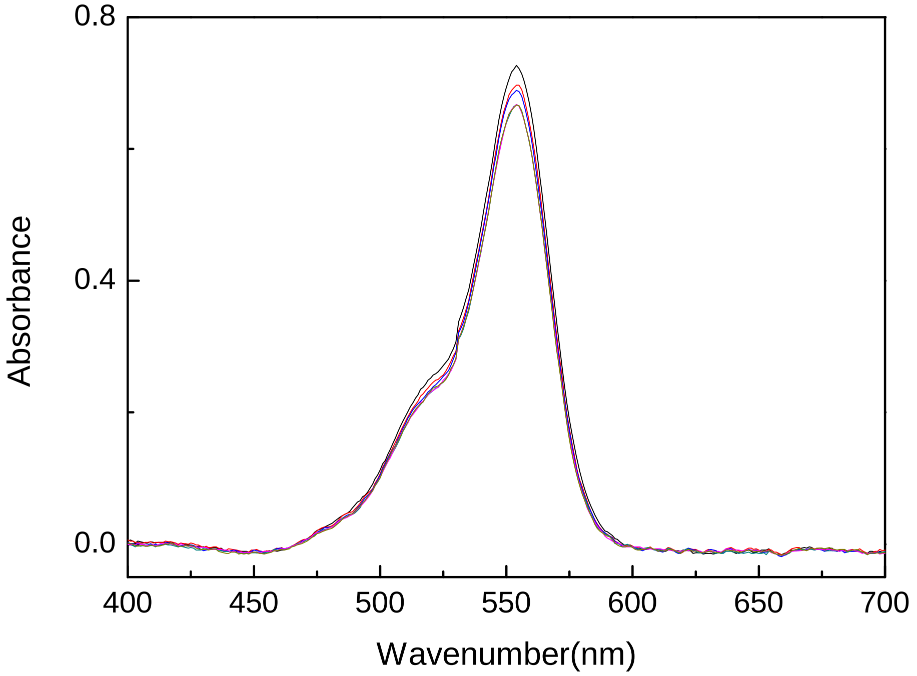

Figure S7 | The temporal evolution of UV spectra of RhB solution (10 mg·L^{-1})

Table S1 | Maximum adsorption capacity of MG on various adsorbents

Adsorbent	Maximum capacity ($mg \cdot g^{-1}$)	Reference
MgO	1205.23	*Mater. Lett.*, 2015, 141, 267
Activated carbon	75.08	*Ind. Eng. Chem. Res.*, 1997, 36, 2207
Zeolitic imidazole framework-67	2430	*Chemosphere*, 2015, 139, 624
Carboxylate group functionalized multi-walled carbon nanotubes	49.45	*J. Mol. Liq.*, 2015, 206, 151
rht anionic metal–organic framework	502	*Chem. Commun.*, 2014, 50, 14674
Boron Nitride Spheres	324	*ACS Appl. Mater. Interfaces*, 2015, 7, 1824
Oxidized multiwalled carbon nanotubes	57.6	*RSC Adv.*, 2015,5, 38939
Hematite–reduced graphene oxide hybrid materials	438.8	*RSC Adv.*, 2015,5, 17336
AA–IA–APT hydrogel	2433	*Chem. Eng. J.*, 2014, 257, 66
Carboxylate functionalized PAN ($PAN/NaOH/NaHCO_3$) nanofibers	1038	*Fiber Polym.*, 2014, 15, 2272
Zeolite–reduced graphene oxide	48.6	*Ind. Eng. Chem. Res.*, 2014, 53, 13711
Sodium alginate-coated Fe_3O_4 nanoparticles	47.84	*Int. J. Bio. Macromol.*, 2014, 69, 447
Acid-activated sintering process red mud	336.4	*Appl. Clay. Sci.*, 2014, 93, 85
Titanium peroxide powder	251.38	*Appl. Surf. Sci.*, 2014, 292, 576
NiO nanoflakes	142.08	*Chem. Eng. J.*, 2014, 239, 141
porous carbon nanosphere	1455	*J. Hazard. Mater.*, 2013, 262, 256
Aminopropyl functionalized magnesium phyllosilicate clay	334.80	*J. Hazard. Mater.*, 2011, 192, 62
Metal–organic framework MIL-100(Fe)	266	*J. Mater. Chem.*, 2012,22, 7449
$ZnO/ZnFe_2O_4$ heterostructures	4983	In this work

Table S2 | Chemical information of the selected dyes

malachite green (MG)	rhodamine (RhB)	methyl orange (MO)
$C_{23}H_{25}ClN_2$	$C_{28}H_{31}ClN_2O_3$	$C_{14}H_{14}N_3SO_3Na$
Mw: 364.92	*Mw*: 479.01	*Mw*: 327.33
Charge: positive	*Charge*: positive	*Charge*: negative
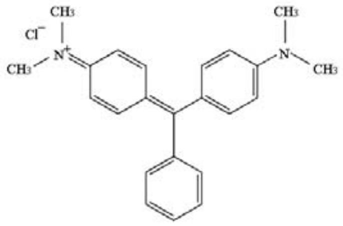	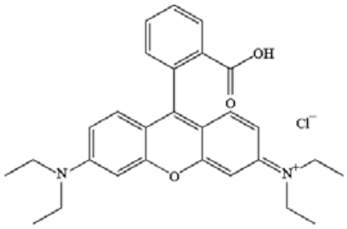	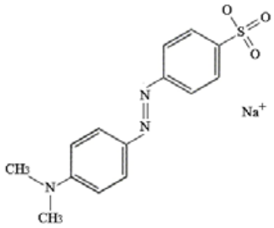
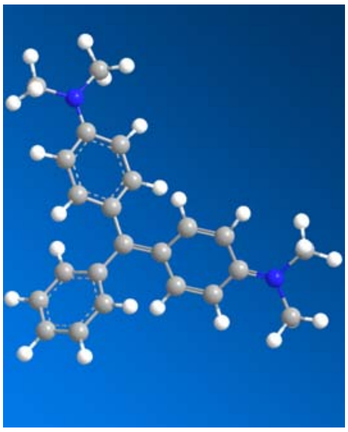	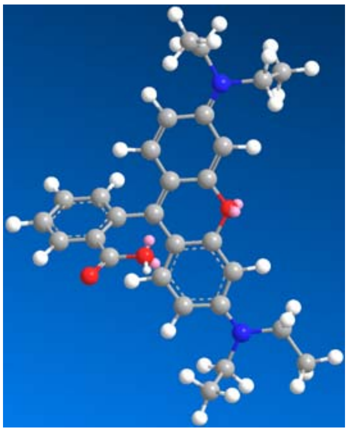	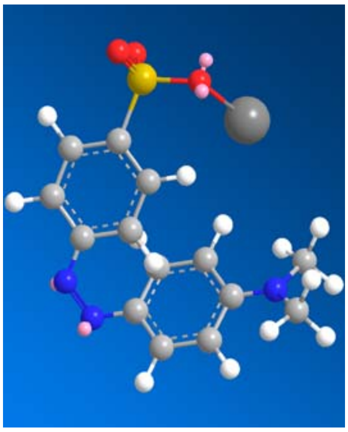

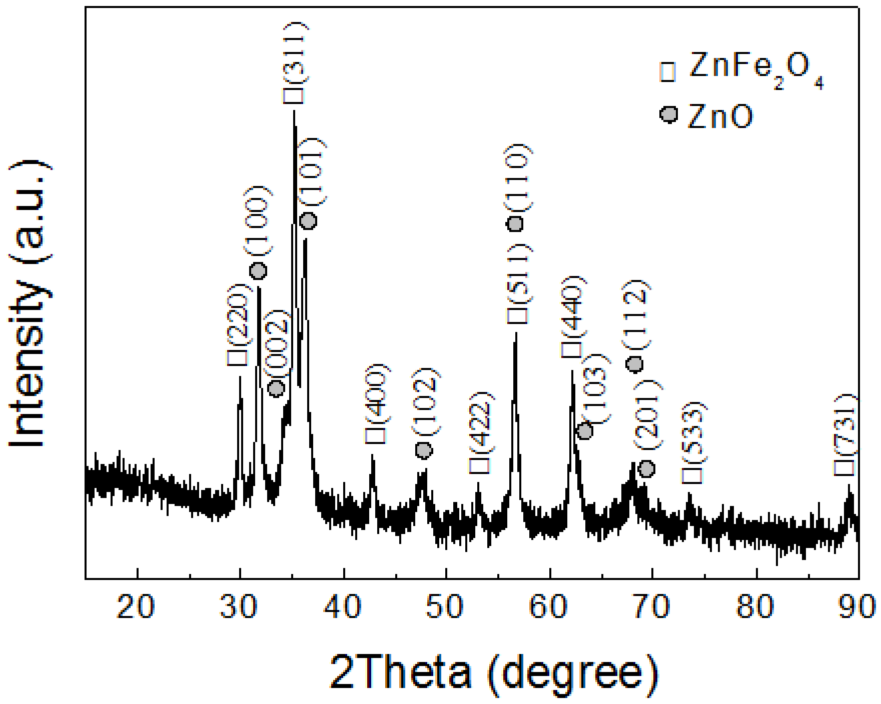

Figure S7 | XRD pattern of the ZnO/$ZnFe_2O_4$ product obtained by thermal annealing at 500 ºC with a ramping rate of 5 ºC·min^{-1}.

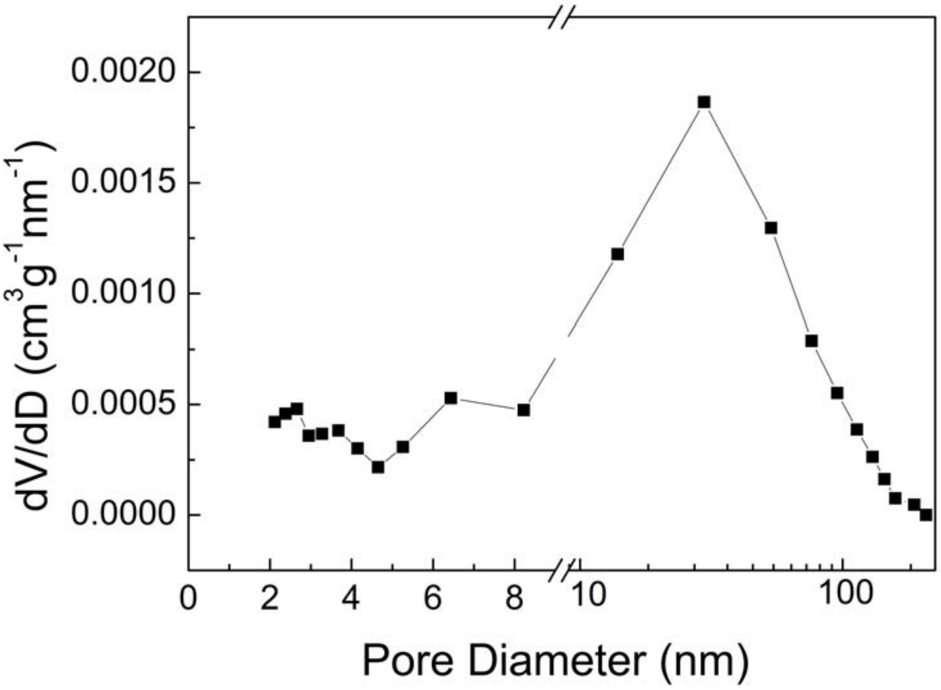

Figure S8 | Pore size distribution of the $ZnO/ZnFe_2O_4$ product obtained by thermal annealing at 500 ºC with a ramping rate of 5 ºC·min^{-1}.

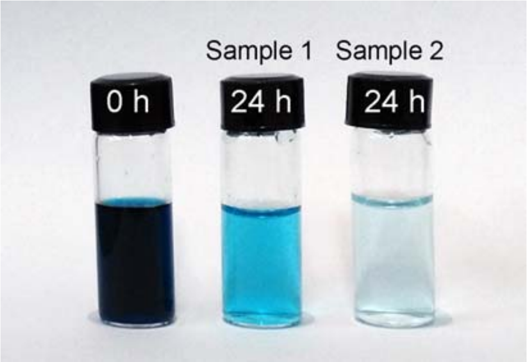

Figure S9 | The colors change of the MG solutions for Sample 1 and Sample 2 after 24 h contact.
